# Exploring karyotype diversity of Argentinian Guaraní maize landraces: Relationship among South American maize

**DOI:** 10.1371/journal.pone.0198398

**Published:** 2018-06-07

**Authors:** María Florencia Realini, Lidia Poggio, Julián Cámara Hernández, Graciela Esther González

**Affiliations:** 1 Facultad de Ciencias Exactas y Naturales, Universidad de Buenos Aires, Departamento de Ecología, Genética y Evolución, Laboratorio de Citogenética y Evolución (LaCyE), Ciudad Autónoma de Buenos Aires, Argentina; 2 Consejo Nacional de Investigaciones Científicas y Técnicas (CONICET), Instituto de Ecología, Genética y Evolución (IEGEBA), Ciudad Autónoma de Buenos Aires, Argentina; 3 Cátedra de Botánica Agrícola, Facultad de Agronomía, Universidad de Buenos Aires, Ciudad Autónoma de Buenos Aires, Argentina; Fred Hutchinson Cancer Research Center, UNITED STATES

## Abstract

In Argentina there are two different centers of maize diversity, the Northeastern (NEA) and the Northwestern (NWA) regions of the country. In NEA, morphological studies identified 15 landraces cultivated by the Guaraní communities in Misiones Province. In the present study we analyzed the karyotype diversity of 20 populations of Guaraní maize landraces through classical and molecular cytogenetic analyses. Our results demonstrate significant intra and inter-populational variation in the percentage, number, size, chromosome position and frequencies of the heterochromatic blocks, which are called knobs. Knob sequence analysis (180-bp and TR-1) did not show significant differences among Guaraní populations. B chromosomes were not detected, and abnormal 10 (AB10) chromosomes were found with low frequency (0.1≥*f* ≤0.40) in six populations. Our results allowed karyotypic characterization of each analyzed population, defining for the first time the chromosomal constitution of maize germplasm from NEA. The multivariate analysis (PCoA and UPGMA) of karyotype parameters allowed the distinction between two populations groups: the Popcorn and the Floury maize populations. These results are in agreement with previously published microsatellite and morphological/phenological studies. Finally, we compared our karyotype results with those previously reported for NWA and Central Region of South America maize. Our data suggest that there are important differences between maize from NEA and NWA at the karyotype level, supporting the hypothesis that there are two pathways of input of South America maize. Our results also confirm the existence of two centers of diversification of Argentinian native maize, NWA and NEA. This work contributes new knowledge about maize diversity, which is relevant for future plans to improve commercial maize, and for conservation of agrobiodiversity.

## Introduction

The Northern region of Argentina is one of the southernmost areas of maize landraces cultivation, where Cámara Hernández *et al*. [1] described 51 morphological native landraces In this region, there are two different centers of diversity: the highland region or Northwestern (NWA) and the Mesopotamic and Chaco plains or Northeastern (NEA) [2]. In the NEA region, 23 native maize landraces have been described, of which 15 are cultivated by the Guaraní communities from the subtropical forests in Misiones province. These landraces have agronomic characteristics that can greatly enrich current agriculture [1, 3]. Analysis of molecular markers and morphological/phenological parameters suggest that these landraces can be divided into different gene pools, corresponding with the type of kernels, the Floury and the Popcorn landraces [3, 4].

Previous studies in lines and landrace of maize from Argentina have shown that the genome size ranges from 4.4 to 6.9 pg [5–8]. Realini *et al*. [7] reported a 2C DNA variation from 4.62 to 6.29 pg in 20 Guaraní populations, revealing significant differences among them. In the *Zea* genus, the variation in DNA content is mainly due to differences in the amount of heterochromatin at distal chromosome blocks called knobs, as well as the presence of B chromosomes [5,9]. Furthermore, there are differences in the amount of interspersed DNA, such as retrotransposon families [10].

Variations in the number, size and position of knobs were reported in American maize landraces [5, 6, 7, 8, 11, 12, 13]. Kato 1976 [11] reported that knobs could be found in 34 different chromosomal positions; a knob may be present or absent at these positions, and its presence and size is a heritable trait. Due to their conserved character, knobs have played an important role in studying the origin and relationships among races of American maize [12]. Knobs consist mainly of highly repeated *tandem* arrangements of two families of satellite-type repeat sequences of 180-bp and 350-bp (TR-1). Both sequences are repeated thousands to millions of times in different proportions relative to each other, forming the different types of knobs; those knobs formed exclusively by 180-bp or TR-1 repeats, and those formed by both sequences in different ratios [14–16]. The knobs sequence composition has been also used to characterize maize landraces [6, 8,17]. FISH experiments using simultaneously 180-bp and TR-1 as probes, revealed the sequence composition of each knob in NWA landraces and 12 Guaraní population from NEA [6,8,17]. In NWA landraces the proportion of knobs that hybridized with the 180-bp were positively correlated with cultivation altitude in contrast with the proportion of knobs that hybridized with the TR-1 that were negatively correlated [18]. The joint analyses of different karyotype parameters (morphology and chromosome size, number, size and position of knob and frequency of B chromosomes), including the knob sequence composition, allowed the cytogenetic characterization of the NWA landraces [6].Some cytological parameters were preliminary described in Guaraní populations to further explore their relationship with the intra-specific genome size variation [7]. Therefore, there is not a comprehensive study that allows the karyotype characterization of NEA maize.

Another karyotypic parameter that was used for maize landraces characterization is the presence and frequencies of B chromosomes (Bs). Maize Bs are widely distributed and large intra- and inter-populational differences in their numbers and frequencies have been reported in NWA landraces [5, 6, 13, 18, 19]. Because Bs have not been previously detected in Guaraní landraces from NEA [20], exhaustive analysis of their occurrence will be a significant contribution to understanding the role of these chromosomes in the diversification of maize landraces. Moreover, the presence of abnormal chromosome 10 (AB10) was also reported and used for cytogenetic characterization of maize and teosinte landraces from America [12, 21]. AB10 is characterized for a knob on its long arm that have about the size of their short arm. Maize neocentromeres observed during meiotic anaphases are associated with AB10 [22–24]. Until now, AB10 chromosomes have not been reported in Argentinian landraces.

McClintock *et al*. 1981 [12], based on knobs chromosome constitution of American landraces, suggested that different types of maize were introduced early at two initial centers of cultivation: Northern South America and the Central Andean highlands. They proposed that maize germplasm from the Northern South America region had a vast influence on the races found in the Caribbean Islands and on those in Eastern South America, whereas races from the Andean Center spread extensively throughout the American Southwest. Using microsatellites analysis, Lia *et al*. 2009 [25] established the affiliation between NWA landraces and Andean complex maize. Recently, an extensive analysis of molecular markers determined the relationship between NEA landraces and those of American Continent lowlands, revealing the existence of three different genetic groups: Tropical Lowland, NEA Flourys and NEA Popcorns [4].

The aim of this work was to analyze the intra- and inter-populational karyotypic variability of Guaraní maize landraces from NEA, to study the chromosome positions, frequencies and sequences composition of knobs, and to define for the first time the chromosomal constitution of this Argentinian maize germoplasm. Our karyotype parameters analysis allowed us to explore the relationships among NEA maize landraces and their affiliation with NWA landraces and with those cultivated in the vicinity regions of lowland South America. This work contributes to knowledge of maize global diversity, an indispensable requirement for its integration into future plans for the improvement of commercial maize and for conservation of agrobiodiversity.

## Material and methods

### Plant materials

Twenty Guaraní maize populations from Northeastern Argentina (NEA) were collected from Guaraní farmers in Misiones Province, Argentina (Table 1). The specimens were deposited at the seed bank of the Vavilov Laboratory, FA-UBA.

**Table 1:** Local names, voucher numbers and collection sites and altitude of cultivation.

Maize landraces (Local Names)	Voucher Numbers (VAV)	Collection sites	Altitude (m.a.s.l)
**Azul** **(Avatí Ovy)**	**6564**۸	Aldea Její, Depto. Guaraní.	329
	**6857**۸	Aldea Yvy Pytá, Ruiz de Montoya, Depto. Libertador General San Martín.	215
**Amarillo Ancho (Avatí ju)**	**6569**۸	Aldea Pozo Azul, Depto. Eldorado.	211
**Amarillo Angosto (Avatí Mitaí)**	**6556**۸	Aldea Guavyra Pory, Paraje Paraíso, Depto. Eldorado.	535
**Blanco Ancho** **(Avatí Morotí)**	**6560**۸	Aldea Chiripa Guaraní, Pindó Poty, Depto. Guaraní.	329
**Blanco Angosto** **(Avatí Para´i)**	**6574**۸	Aldea Mirِí, Depto. Guaraní.	98
**Overo** **(Avatí Pará)**	**6559**۸	Paraje Paraíso, Depto. San Pedro.	525
	**6823**۸	Aldea Yvy Pytá, Ruiz de Montoya, Depto. Libertador General San Martín.	215
**Rosado** **(Avatí Yui)**	**6565**۸	Colonia Její, Depto. Guaraní.	329
**Variegado** **(Avatí Tove)**	**6557**۸	Paraje Paraíso, Depto. San Pedro.	525
**Colorado** **(Avati Pyta´í)**	**6837**^**#**^	Aldea Perutí, El alcázar, Depto. Libertador General San Martín.	591
	**6573**^**#**^	Aldea Mirí, Depto. Candelaria (Municipio de Sta. Ana).	98
**Tupí Amarillo (Avatí Tupí)**	**6563**^**#**^	Aldea Pindó Poty, Depto. Guaraní.	329
**Tupí Blanco** **(Avatí Tupí)**	**6592**^**#**^	Pozo Azul, Depto. San Pedro.	211
**Pipoca Amarillo**	2011/09*	Aldea Pozo Azul, Depto. Eldorado.	211
	6568*	Aldea Pozo Azul, Depto. Eldorado.	211
**Pipoca Colorado**	6607*	Aldea Alecrín, Depto. Eldorado.	211
	6567*	Aldea Pozo Azul, Depto. Eldorado.	211
**Pororó Chico** **(Avatí Pororó)**	6575*	Aldea Guavyra Pory, Paraje Paraíso, Depto. San Pedro.	525
**Pororó Grande** **(Avatí Pororó)**	6562*	Aldea Pindó Poty, Depto. Guaraní.	329

Ref.

* Corneal grains (Popcorn-Pc)

۸ floury grains (Floury-F)

# floury grains with corneal periphery (F-Pc).

In Table 1, the type of grain is indicated for each population, Floury (F), Floury with corneal periphery (F-Pc) and Popcorn (Pc). The term population is used here to refer to a set of individuals belonging to a morphological race, which are cultivated by a farmer or family group in a location.

### Methods

Seeds were germinated at 28°C for 2–3 days in Petri dishes containing wet filter paper. Primary root tips, 0.5–1 cm in length, were pre-treated with 8-hydroxiquinoline (0.02 M, Sigma) for 5 h at room temperature. Then they were fixed in 3: 1 (ethanol: acetic acid) and stored at 4°C until use.

### Chromosomal preparations

Mitotic metaphase preparations were performed in 20 maize populations. Fixed root tips were treated with an enzymatic solution (2% cellulose, Onozuka R10 Merck, and 20% Pectinase, Sigma P4716) for 1 h at 37°C. After freezing to remove the coverslips, the slides were air-dried and stored at 4°C until use.

### DAPI staining (4′,6-Diamidino-2-phenylindole)

DAPI identifies regions with highly repeat DNA sequences rich in A-T such as knobs, which are visualized as DAPI positive bands (DAPI +).This technique was carried out according to Sumner 1990 [26]. Slides were washed in McIlvaine buffer (citric acid–NaHPO buffer, pH 7), and then stained with 2 μg/ml DAPI (Sigma-Aldrich, St. Louis, MO, USA). After staining, preparations were briefly washed. Slides were mounted in Mcllvaine buffer and sealed with rubber solution.

### DNA probes

For FISH analysis, the maize knob sequences, 180-bp, TR-1(350 bp) and rDNA 5S and 18S were obtained from GenBank (http://www.ncbi.nlm.nih.gov/), the centromeric sequences of maize CentC probe was designed by a CentC *consensus* repeats provided by Dr. Kelly Dawe of the University of Georgia, USA. The primers were designed using the Primer3 program (version 0.6) provided by the Whitehead Institute for Biomedical Research & Howard Hughes Medical Institute, USA (http://bioinfo.ut.ee/primer3/). These sequences were isolated and amplified from total genomic DNA of maize by polymerase chain reaction (PCR) methods [27]; The PCR cycling conditions were 4 min at 94° C, followed by 30 cycles of 1 min at 94° C, 1 min at 50–60° C (annealing changing according to primer Tm), 1 min at 72° C and a final extension at 72° C for 7 min Cycling was done in an Eppendorf Mastercycler (Eppendorf, Hamburg, Germany). The probes were biotin and digoxigenin-labelled, by PCR, using fluorescently conjugated nucleotide 16-biotin-dUTP (Sigma) and 11-digoxigenin-dUTP (Roche, Mannheim, Germany).

### Fluorescence *in situ* hybridization

The FISH procedure was performed as Realini *et al*. [7], with minor modifications. Slide preparations were incubated in 100 μg mL^-1^ of RNAse (Sigma) in 2× saline sodium citrate (2 × SSC) for 1 h at 37°C in a humidified chamber and washed three times in 2 × SSC for 5 min each at room temperature. The slides were post-fixed in freshly prepared 4% (w/v) paraformaldehydein (Fluka) for 10 min and then washed in 2 × SSC for 15 min at room temperature. Then, the preparations were dehydrated in a graded ethanol series and air-dried. To each preparation was added 30 μL of the hybridization mixture, which contained 50 ng of each labelled probe. The hybridization mixture was denatured for 15 min at 75°C. The slides were placed on a thermocycler at 75°C for 7 min, 45°C for 10 min and 38°C for 10 min. Then the slides were incubated overnight at 37°C. Post-hybridization washes were carried out [7]. The slides were incubated in the detection buffer containing 2.5% bovine serum albumin and the corresponding detection antibodies Streptavidine-CY3 conjugate (Sigma) or antidigoxigenin-fluorescein isothiocyanate-FITC (Sigma), for 1 h at 37°C. Slides were washed three times in 4 × SSC/Tween buffer for 10 min at room temperature, counterstained with 1 μg mL^-1^ of DAPI in 4 × SSC/Tween buffer for 40 min at room temperature and mounted in Vectashield antifade solution (Vector Laboratories, Burlingame, CA, USA). Slides were examined with a Carl Zeiss Axiophot epifluorescence microscope (Carl Zeiss, Germany), with appropriate Carl Zeiss filters coupled with a Leica DC 250 digital camera and with an image analyzer Leica IM 1000. The location of hybridization signals and DAPI positive bands were based on the observation of at least 20 complete metaphases for each analyzed individual.

### Karyotype parameters

For characterization and detection of Bs and AB10 chromosomes, 4 to 9 individuals were studied for each population sampled. At least 20 similar condensed metaphases for each individual were chosen, photographed and used for karyotype parameters estimation. The identification of each pair of chromosomes was based on the maize chromosomal morphology described by McClintock *et al*. 1981 [12], as well as on ribosomal and centromeric sequences mapping by FISH. Karyotype parameters were determined using MicroMeasure V.3.3 (www.colostate.edu/Depts/Biology/MicroMeasure). Centromeric index (CI) and total chromosome length (TCL) were estimated. The symmetry of karyotypes was studied using the A_1_ [28], CV_CL_, CV_CI_ [29] and M_CA_ indexes [30]. *Consensus* idiograms were performed for each Guaraní maize landrace using Adobe Photoshop CS3 version 10.0. Chromosome position, size and appearance frequencies of each knob were indicated. The knob frequencies were represented in a histogram for each population. In the histograms and idiograms, the most frequent (*f*≥ 0.6) positions were indicated with black bars/block, while the least frequent positions (*f*< 0.6) were indicated with white bars/blocks. For all data *consensus* idiogram and frequencies appearance of knob histogram were constructed, representing the karyotypes from Guaraní maize from NEA. Knob frequencies appearance of knob histograms were constructed for the populations with different type of grains, Floury (F), Floury grains with corneal periphery (F-Pc) and Popcorn (Pc) maize, respectively.

### Statistical analysis

Correlation analyses were performed using the Spearman coefficient. The variables correlated were: percentages of heterochromatin, number of knobs, total chromosome length (TCL) and asymmetric indexes (A_1_, CV_CL_, CV_CI_ and M_CA_). A linear regression analysis was performed between percentages of heterochromatin and the number of knobs. These statistical analyses were considered significant at P-values ≤0.05.

For each continuous karyotype variable (percentage of heterochromatin, numbers of knobs, TCL and CV_CI_, CV_CL_, M_AC_ and A_1_ indexes), an analysis of variance (ANOVA) was performed to test the karyotype differences among populations with different types of grain. The variance was modulated with VarIdent. The seven ANOVAs were applied with a global level of 0.05 using the Bonferroni´s method, so the differences were considered significant at P-values ≤0.007. These analyses were followed by multiple comparison Fisher’s least significant difference test [31].

A principal coordinated analysis (PCoA) and a UPGMA multivariate analysis were performed using the continuous and binary (presence/absence of knob positions) karyotype variables. The 6S and 6-Sat knob positions were excluded from these analyses if they were either always absent or always present in the studied individuals, respectively. In both analyses the data was standardized. Since a mixture of variable types (binary and continuous) was used, Gower distance-based similarity matrix was employed [32]. For the PCoA analysis Biplots, two-dimensional graphs, axis1 *vs*. axis 2, axis1 *vs*. axis 3 and axis 1 *vs*. axis 4 were represented. In these graphs the contours correspond to 80% prediction ellipses for individuals belonging to F and Pc maize.

All statistical analyses were performed in 17 Guaraní populations using the program Infostat, FCA, National University of Córdoba [33]. For the unbalanced design, the R package [34] was used.

## Results

### Karyotype analysis

**Fig 1:**
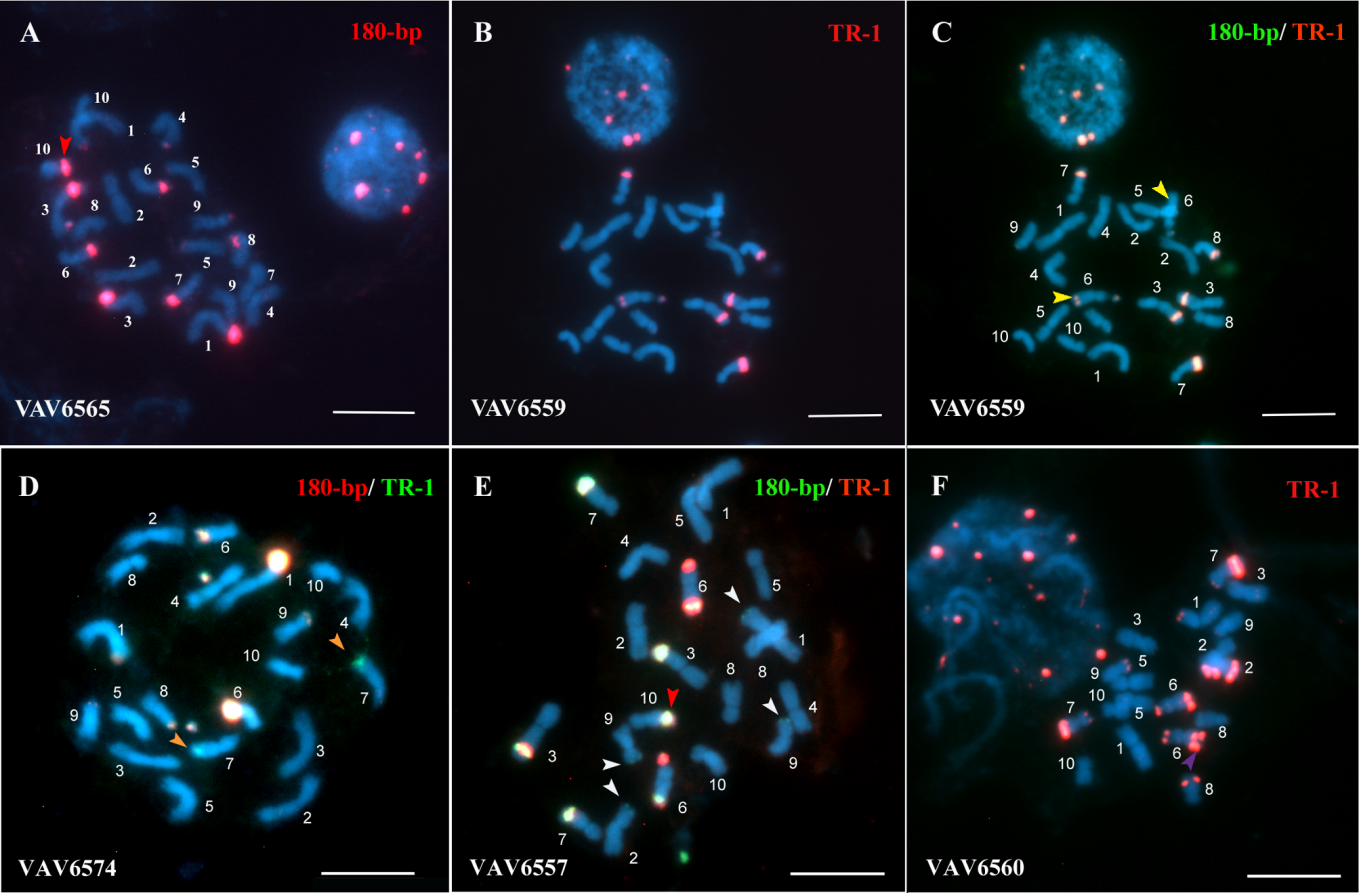
Characterization of Floury Guaraní maize metaphase chromosomes by FISH with knobs probes. (A-F) merge DAPI (blue) / FISH. (A) VAV6565, Rosado. (B) and (C) VAV6569, Overo. (D) VAV6574, Blanco Angosto. (E) VAV6557, Variegado. (F) VAV6560, Blanco Ancho. The probes were labeled with digoxigenin and biotin, and revealed with antidigoxigenin-FITC (green) and Cy3 (red), respectively. Ref. The red arrowhead indicates a chromosome with AB10 morphology. The yellow arrowheads show heterozygosis for the 6L knob chromosome position. The white arrowhead indicates knobs hybridized only with 180-bp sequence. The orange arrowhead indicates knobs hybridized only with TR-1 sequence. The numbers indicates the chromosomal pairs. Scale bars = 10 μm.

**Fig 2:**
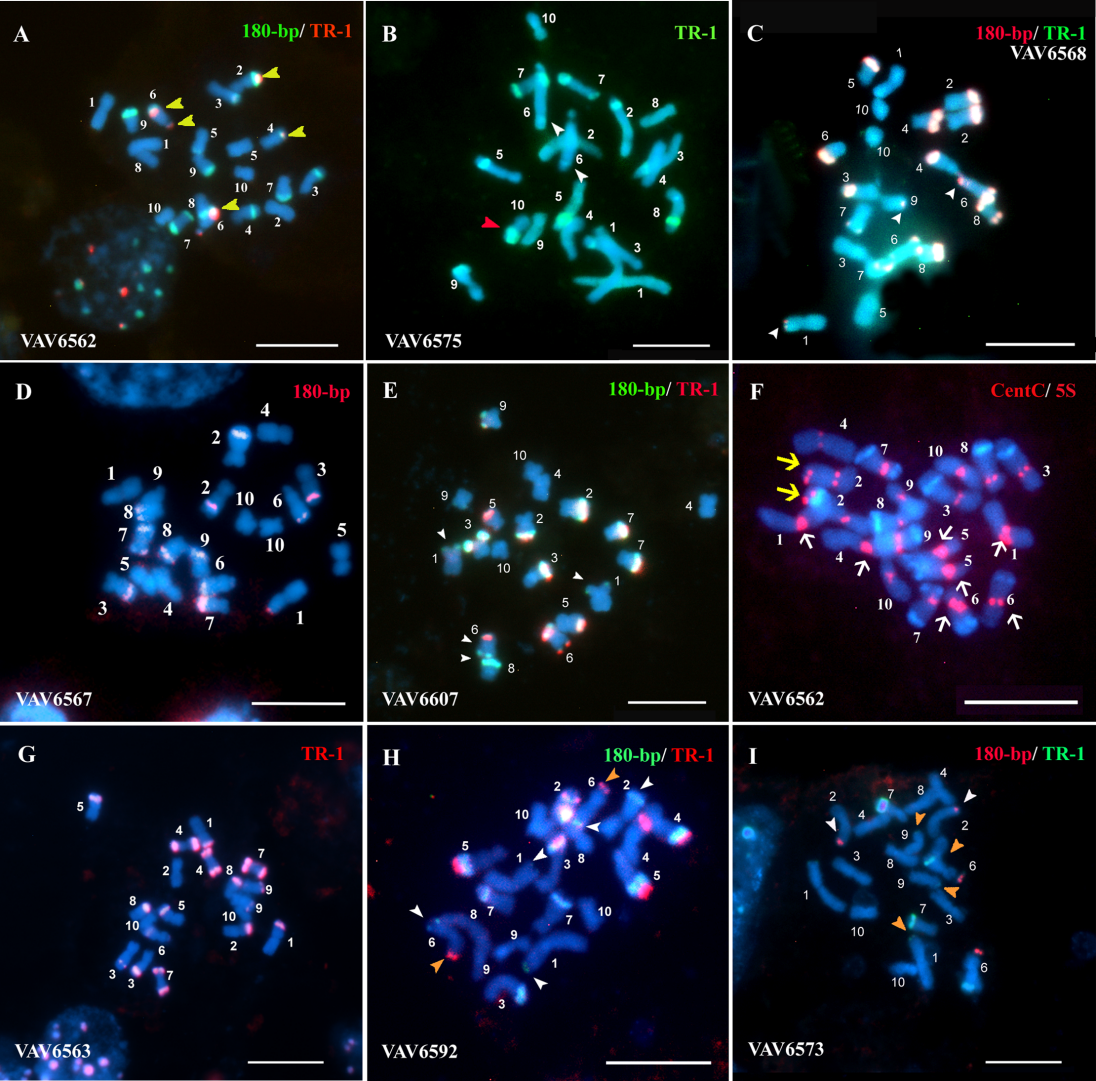
Characterization of Popcorn and F-Pc Guaraní maize metaphase chromosomes by FISH with knobs, CentC and 5S rDNA probes. (A-I) merge DAPI (blue) / FISH. (A) and (F) VAV6562, Pororó Grande. (B) VAV6575, Pororó Chico. (C) VAV6568, Pipoca Amarillo. (D) VAV6567, Pipoca Colorado. (E) VAV6607, Pipoca Colorado. (G) VAV6563, Tupí Amarillo. (H) VAV6592, Tupí Blanco. (I) VAV6567, Colorado. Ref. The white arrowhead indicates knobs hybridized only with 180-bp sequence. The orange arrowhead indicates knobs hybridized only with TR-1 sequence. The red arrowhead indicates a chromosome with AB10 morphology. The yellow arrow indicates 5S rDNA hybridization signal. The white arrows indicate the most intense and / or higher hybridization signals of CentC sequence. The numbers indicate the chromosomal pairs. Scale bars = 10 μm.

The DAPI chromosome banding and FISH revealed wide variation in number (between 8 and 23) and size (from small-S_K_ to large-L_K_) of knobs / DAPI^+^-FISH^+^ bands among 20 studied Guaraní maize populations. Knobs were observed in 20 different chromosomal positions (1S, 1L, 2S, 2L, 3S, 3L, 4S, 4L, 5S, 5L, 6-Sat, 6L_2_, 6L_3_, 7S, 7L, 8S, 8L, 9S, 9L and 10L), with inter-populational differences in their frequencies (Table 2). The more resolutive FISH assays allowed detecting knobs more accurately than DAPI banding, particularly in those cases where the knob sequences were in low copy numbers. The populations presented heterozygosity for the presence / absence of the different positions and size of knobs (Figs 1 and 2). As was previously reported [7], the more exhaustive study of VAV6563 corroborated that this population present the largest number of knobs in 13 different karyotype positions, all with high frequencies, except for 3S. The populations VAV6573, VAV6564, and VAV6569 showed between 11 and 16 different chromosomal positions, with seven (1S, 3L, 6-Sat, 6L, 7L, 8L, 9S), six (1S, 4S, 6-Sat, 6L, 7L, 9S) and four (1S, 3L, 6-Sat, 6L) positions with high frequencies, respectively. Only the population VAV6560 presented two knob positions on the large arm of chromosome 6, 6L_2_ and 6L_3_ (Fig 1F). Chromosomes with AB10 morphology were observed with low frequency (0.1≥ *f* ≤0.40) in VAV6565, VAV6575, VAV6607, VAV6564, VAV6568 and VAV6557 populations (Figs 1A, 1E and 2B). B chromosomes were not detected in any studied individual from the 20 studied populations.

**Table 2:** Range of percentages of heterochromatin, range of number of knobs, chromosomal positions of knobs, means of asymmetry indexes (± SD).

Maize landraces/ populations (VAV)	Range of the percentage of knob heterochromatin ($\bar{X}$ ± SD)	Range of the numbers of knobs ($\bar{X}$ ± SD)	Chromosome positions of knobs	Asymmetric indexes ($\bar{X}$ ± SD)
**Amarillo Ancho** **VAV6569**	6.00% - 9.19% (7.78% ± 1.43)	10–16 (12.50 ± 2.35)	**1S**, 2L, 3S, **3L**, 4L, 5S, 5L, **6-Sat**, **6L**,7S, 7L, 8S, 8L, 9S, 9L, 10L	**CV**_**CI**_ = 14.03% ± 2.34 **CV**_**CL**_ = 22.02% ± 2.91 **A**_**1**_ = 0.32 ± 0.06 **M**_**AC**_ = 20.61% ± 4.14
**Amarillo Angosto** **VAV6556**	5.91% - 10.32% (7.07% ± 1.57)	10–17 (11.43 ± 3.15)	**1S**, **3L**, 4S, 4L, 5L, **6-Sat**, **6L**, 7S, 7L, 8L, **9S**, 9L	**CV**_**CI**_ = 13.18% ± 2.09 **CV**_**CL**_ = 24.75% ± 5.06 **A**_**1**_ = 0.29 ± 0.07 **M**_**AC**_ = 17.23% ± 4.06
**Azul** **VAV6564**	5.57% - 11.51% (7.03% ± 2.59)	8–19 (12.40 ± 5.18)	**1S**, 2S, 3S, 3L, **4S**, 4L, 5S, 5L, **6-Sat**, **6L**, **7L**, 8L, **9S**, 10L	**CV**_**CI**_ = 16.49% ± 4.74 **CV**_**CL**_ = 22.98% ± 4.64 **A**_**1**_ = 0.33 ± 0.60 **M**_**AC**_ **=** 19.98% ± 3.06
**Azul** **VAV6857**	11% - 13%	14–16	1S, 1L, 2S, 2L, 3L, 4S, 4L, 5L, 6-Sat, 6L, 7S, 7L, 8S, 8L, 9S	**CV**_**CI**_ = 14.50% ± 0.02 **CV**_**CL**_ = 21.19% ± 0.01 **A**_**1**_ = 0.29 ± 0.01 **M**_**AC**_ = 18.00% ± 0.00
**Blanco Ancho** **VAV6560**	5.21% - 10.66% (7.34% ± 2.03)	9–17 (12.11 ± 2.67)	**1S**, 2S, 2L, 3S, 3L, 4S, 4L, 5L, **6-Sat**, **6L**_**3**_, 6L_2_,7S, **7L**, 8S, 8L, **9S**, 9L, 10L	**CV**_**CI**_ = 15.48% ± 1.36 **CV**_**CL**_ = 22.94% ± 3.25 **A**_**1**_ = 0.33 ± 0.05 **M**_**AC**_ = 20.06% ± 3.40
**Blanco Angosto** **VAV6574**	5.06% - 8.51% (6.37% ± 1.24)	8–11 (9.83 ± 0.98)	**1S**, 2L, 3L, 4L, **6-Sat**, **6L**, **7L**, 8L, **9S**, 9L	**CV**_**CI**_ = 15.63% ± 3.14 **CV**_**CL**_ = 25.36% ± 2.29 **A**_**1**_ = 0.35 ± 0.03 **M**_**AC**_ = 22.01% ± 2.67
**Overo** **VAV6559**	5.51% - 8.60% (7.19% ± 1.32)	9–13 (11.50 ± 1.64)	1S, 2L, 3S, **3L**, 4L, 5S, **6-Sat**, **6L**, **7L**, 8S, **8L**, 9S, 9L	**CV**_**CI**_ = 15.85% ± 1.90 **CV**_**CL**_ = 21.41% ± 1.34 **A**_**1**_ = 0.33 ± 0.03 **M**_**AC**_ = 20.32% ± 2.43
**Overo** **VAV6823**	*ca*. 8%	12–15	1S, 2L, 3L, 4L, 6-Sat, 6L, 7S, 7L, 8L, 9S, 9L	**CV**_**CI**_ = 16.45% ± 0.03 **CV**_**CL**_ = 26.46% ± 0,03 **A**_**1**_ = 0,33 ± 0.07 **M**_**AC**_ = 20.83 ± 0.05
**Rosado** **VAV6565**	6.36% - 11.02% (8.34% ± 1.78)	11–16 (13.33 ± 1.97)	**1S**, 2S, 2L, **3L**, 4S, 4L, 5S, 5L, **6-Sat**, **6L,** 7S, 7L, 8S, 8L, **9S**, 9L, 10L	**CV**_**CI**_ = 13.60% ± 0.83 **CV**_**CL**_ = 24.87% ± 4.57 **A**_**1**_ = 0.32 ± 0.03 **M**_**CA**_ = 20.32% ± 2.16
**Variegado** **VAV6557**	5.30% - 8.63% (7.74% ± 1.77)	10–15 (12.40 ± 5.18)	**1S**, 2S, 3S, **3L**, 4S, 4L, 5S, 5L, **6-Sat**, **6L**, **7L**, 8L, **9S**, 10L	**CV**_**CI**_ = 14.10% ± 2.19 **CV**_**CL**_ = 22.94% ± 2.78 **A**_**1**_ = 0.37 ± 0.02 **M**_**AC**_ = 23.18% ± 1.47
**Colorado** **VAV6837**	6.78% - 10.07% (8.43% ± 1.44)	11–15 (13.00 ± 1.83)	**1S**, 3L, 4L, 5S, 5L, **6-Sat**, **6L**, 7S, **7L**, 8S, **8L**, **9S**, 9L	**CV**_**CI**_ = 16.65% ± 3.32 **CV**_**CL**_ = 21.62% ± 1.74 **A**_**1**_ = 0.33 ± 0.09 **M**_**AC**_ = 23.49% ± 3.38
**Colorado** **VAV6573**	7.27% - 10.88% (8.56% ± 1.50)	10–14 (12.20 ± 1.48)	**1S**, 2L, 3S, **3L**, 4L, **6-Sat**, **6L**, **7L**, **8L**, **9S**, 9L	**CV**_**CI**_ = 16.99% ± 2.46 **CV**_**CL**_ = 20.75% ± 2.13 **A**_**1**_ = 0.30 ± 0.04 **M**_**AC**_ = 18.96% ± 2.46
**Tupí Amarillo** **VAV6563**	13.90% - 20.02% (16.71% ± 2.52)	17–23 (19.75 ± 2.50)	**1S**, **2L**, 3S, **3L**, **4S**, **4L**, **5L**, **6-Sat, 6L**, **7S**, **7L**, **8L**, **9L**	**CV**_**CI**_ = 16.57% ± 1.99 **CV**_**CL**_ **=** 22.31% ± 3.03 **A**_**1**_ = 0.34 ± 0.05 **M**_**AC**_ **=** 21.66% ± 3.54
**Tupí Blanco** **VAV6592**	12.15% - 14.38% (13.31% ± 0.93)	14–19 (16.50 ± 2.08)	**1S**, **2L**, **3L**, 4S, **4L**, **5L**, **6-Sat**, **6L**, 7S, **7L**, **8L**, 9S	**CV**_**CI**_ = 16.26% ± 1.54 **CV**_**CL**_ **=** 23.51% ± 1.33 **A**_**1**_ = 0.32 ± 0.01 **M**_**AC**_ **=** 20.06% ±0.40
**Pipoca Amarillo** **VAV2011/09**	10.24% - 16.66%	12–17	1S, 2S, 3L, 4L, 5L, 6-Sat, 6L, 7S, 7L, 8L, 10L	**CV**_**CI**_ = 16.47% ± 0.01 **CV**_**CL**_ = 29.49 ± 0.02 **A**_**1**_ = 0.38 ± 0.07 **M**_**AC**_ = 24.96% ± 0.06
**Pipoca Amarillo** **VAV6568**	9.91% - 15.80% (12.82 ± 2.12)	14–19 (15.25 ± 2.87)	**1S**, **2L**, **3L**, **4L**, 5L, **6-Sat, 6L**, **7S**, **7L**, 8S, **8L**, **9S**, 9L, 10L	**CV**_**CI**_ = 16.23% ± 4.26 **CV**_**CL**_ = 24.22% ± 2.96 **A**_**1**_ = 0.32 ± 0.03 **M**_**AC**_ = 20.63% ± 2.74
**Pipoca Colorado** **VAV6567**	9.04% - 13.46% (11.62% ± 1.69)	14–17 (15.60 ± 1.52)	**1S, 2L, 3L, 4L,** 5S, **5L, 6-Sat**, **6L, 7S, 7L, 8L, 9S, 9L**	**CV**_**CI**_ = 18.83% ± 4.97 **CV**_**CL**_ = 24.07% ± 4.86 **A**_**1**_ = 0.36 ± 0.06 **M**_**AC**_ = 23.90% ± 5.05
**Pipoca Colorado** **VAV6607**	9.72% - 15.64% (12.43% ± 2.17)	15–21 (17.83 ± 2.40)	1S, **2L, 3L, 4S, 4L,** 5S, **5L, 6-Sat, 6L, 7L**, **8L**, **9S**, 9L, 10L	**CV**_**CI**_ = 17.05% ± 2.25 **CV**_**CL**_ = 24.04% ± 3.58 **A**_**1**_ = 0.36 ± 0.03 **M**_**AC**_ = 22,96% ± 2,12
**Pororó Chico** **VAV6575**	10.21% - 14.84% (12.38% ± 2.20)	16–18 (17.50 ± 1.00)	**1S**, **2L**, **3L**, 4L, **5L**, **6-Sat**, **6L**, **7S**, **7L**, **8L**, 9S, 9L, 10L	**CV**_**CI**_ = 18.57% ± 5.27 **CV**_**CL**_ = 25.89% ± 4.92 **A**_**1**_ = 0.36 ± 0.06 **M**_**AC**_ = 23.11% ± 4.81
**Pororó Grande** **VAV6562**	10.82% - 15.81% (13.59% ± 1.66)	15–20 (17.00 ± 1.51)	1S, 1L, 2S, **2L**, 3S, **3L**, **4L**, 5L, **6-Sat**, **6L**, **7S**, **7L**, **8L**, 9S, **9L**	**CV**_**CI**_ = 17.83% ± 3.14 **CV**_**CL**_ = 21.53% ± 3.06 **A**_**1**_ = 0.35 ± 0.05 **M**_**AC**_ **=** 22.21% ± 4.05

**Ref.** The most frequent chromosomal positions of knobs (*f*≥ 0.6) are highlighted in bold. SD: standard deviation.

The percentages of heterochromatin were calculated as the percentage of the total chromosomal complement occupied by the knob heterochromatin. A variation range from 5% to 20% was found among populations. For example, the VAV6574 population had the lowest mean number of knobs ($\bar{X}$ = 9.83) and the lowest percentage of heterochromatin ($\bar{X}$ = 6.37%), while the VAV6563 population showed the highest mean number of knobs ($\bar{X}$ = 19.75) and the highest percentage of heterochromatin ($\bar{X}$ = 16.71%). Intra-populational variation in the number of knobs and percentage of heterochromatin was also detected (Table 2). For example, the VAV6564 population varied from 8 to 19 knobs, and the percentage of heterochromatin ranged from 5.57% to 11.51%. A significant positive relationship was found between the number of knobs and percentages of heterochromatin (p≤0.0001, Spearman coefficient (SC) = 0.875, df = 95), and a positive linear relationship was detected between both parameters (Y = -2.97 + 0.94X, r = 0.71, n = 101, F = 239.96, p <0.0001, where Y represents the percentages and X indicates the number of knob).

The CI, TCL and karyotype asymmetry indexes (A_1_, M_AC_, CV_CI_ and CV_CL_) were calculated for each studied individual (Table 2 and S1 Table). The chromosomal asymmetry indexes showed inter- and intra-populational variation (Table 2). These karyotype parameters were correlated with percentages of heterochromatin and numbers of knob, respectively. The CV_CI_ was significantly correlated with percentages of heterochromatin and number of knobs, while TCL, A_1_, M_AC_ and CV_CL_ did not show significant correlation with both karyotype parameters (S2 Table).

### Karyotype differences among Floury, F-Pc and Popcorn populations

The Popcorn and Tupí (F-Pc) populations showed the highest values in number of knobs and percentage of heterochromatin, while the Floury populations showed the lowest values for these two parameters (Table 2). Analysis of variance (ANOVA) performed on the karyotype parameters (number of knobs, percentage of heterochromatin, TCL, A_1_, M_AC_ CV_CI,_ and CV_CL_ indexes) showed significant differences in the number of knobs, percentage of heterochromatin and CV_CI_ index (F _2,14_ = 26.55, P< 0.0001; F _2,14_ = 85.47, p< 0.0001; F _2,14_ = 9.98, p< 0.0022; respectively) among the different types of grain populations. The Pc and F populations showed significant differences in these three karyotype parameters. The F-Pc populations did not show differences with Pc or F populations in the number of knobs and percentage of heterochromatin, but they did show significant differences with F populations in their CV_CI_ index values.

### FISH sequences mapping

Fluorescent *in situ* hybridization (FISH) assays on mitotic metaphases, using 180-bp and TR-1 knob sequences as probes, showed variations in the hybridization signals of the different knobs. In the studied individual, the majority of their knobs exhibited 180-bp and TR-1 signal overlapped (mixed knobs). The knobs that were hybridized with one single knob sequence showed, in general, the 180-bp signal (Figs 1D, 1F, 2B, 2D, 2H and 2I). Although in VAV6592 the majority of knobs were mixed, in some chromosome pairs both signals did not overlapped, the TR-1 was located at different chromosomal positions than 180-bp. This can be observed on the chromosome pairs 4 and 5 in Fig 2H.

The ribosomal sequences 18S and 5S hybridized on the secondary constriction of the short arm of chromosome 6 (6S) and on the long arm of chromosome 2 (2L), respectively, with no differences in intensity of hybridization signals between the analyzed individuals. Hybridization signals from the centromeric sequence CentC allowed a precise identification of chromosomal morphology, and showed variation in intensity and size among chromosomes from the same metaphase and among individuals from different populations (Fig 2F).

### Guaraní landraces chromosomal characterization

In S1 and S2 Figs, the relative size, chromosome position, and the most and least frequent positions of knobs are summarized in representative idiograms for each population. The frequencies for each chromosomal position of knob are shown in histograms (S1 and S2 Figs).

**Fig 3:**
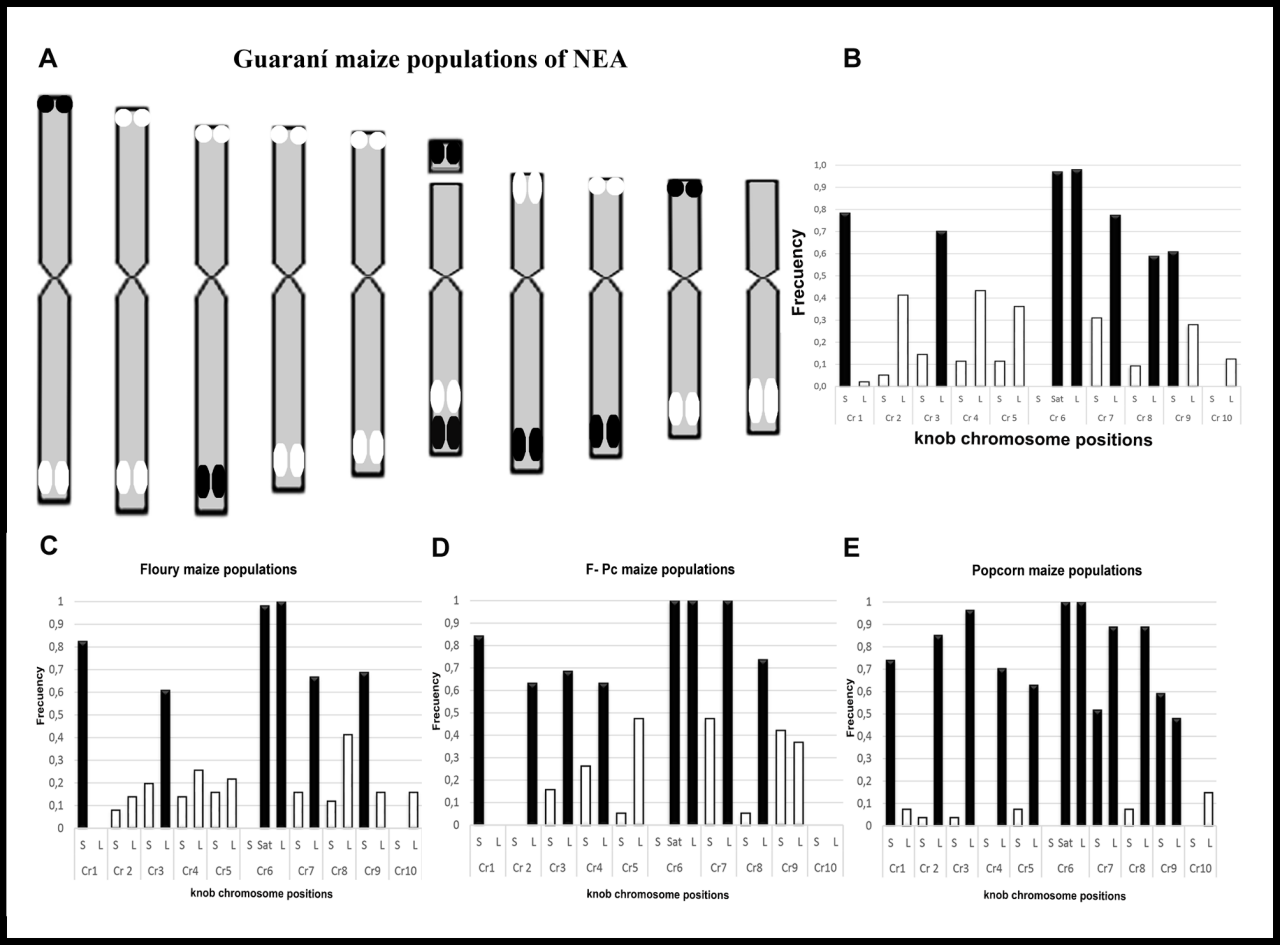
*Consensus* idiogram and knob position histogram of Guaraní maize of NEA, where the position, average size and frequency of each knob are indicated. (A) *Consensus* idiogram of Guaranís maize from NEA. (B) Knob position histogram. (C-E) Knob position histograms for maize population with different types of grains, Floury-F (C), Floury grains with corneal periphery-F-Pc (D) and Popcorn-P populations (E). Ref. The position, average size and knob frequencies are indicated on the idiogram. The average size of the knobs are represented by the size of the bands (S_k_, M_k_ and L_k_). The black blocks/ bars indicate the most frequent positions (*f* ≥0.6). The white blocks/ bars show positions of less frequency (*f*<0.6). The size of each knob was estimated in relation to thechromosome length: small knobs (S_k_) ≤ 10%, medium knobs (M_k_) between 10% and 20%, and large knobs (L_k_) ≥ 20%. Cr: chromosomal pair. L: long arm. S: short arm. Sat: satellite region.

To represent the chromosomal constitution of Guaraní maize from NEA, we constructed a *consensus* idiogram and histogram with data from all analyzed populations (Fig 3A and 3B). The positions 1S, 3L, 6-Sat, 6L, 7L, 8L and 9S were the most frequent (*f* ≥0.60), while 1L, 2S, 3S, 4S, 5S, 8S and 10L were the least frequent (*f ca*. 0.1). In general, it was observed that the knobs on the long arm were medium (M_K_) or large (L_k_), while the knobs located on the short arms were small (S_K_). Fig 3C, 3D and 3F show the histograms of each knob position for the Floury (F), Floury grains with corneal (F-Pc) and Popcorn (P) populations, respectively. We found that the F populations have only 6 knobs chromosome positions with high frequencies (1S, 3L, 6-Sat, 6L, 7L, 9S), whereas F-Pc and Pc populations have a higher number of knobs positions with high frequencies (1S, 2L, 3L, 4L, 6L, 6L, 7L, 8L and 1S, 2L, 3L, 4L, 5L, 6-Sat, 6L, 7L, 8L, 9S, respectively).

**Fig 4:**
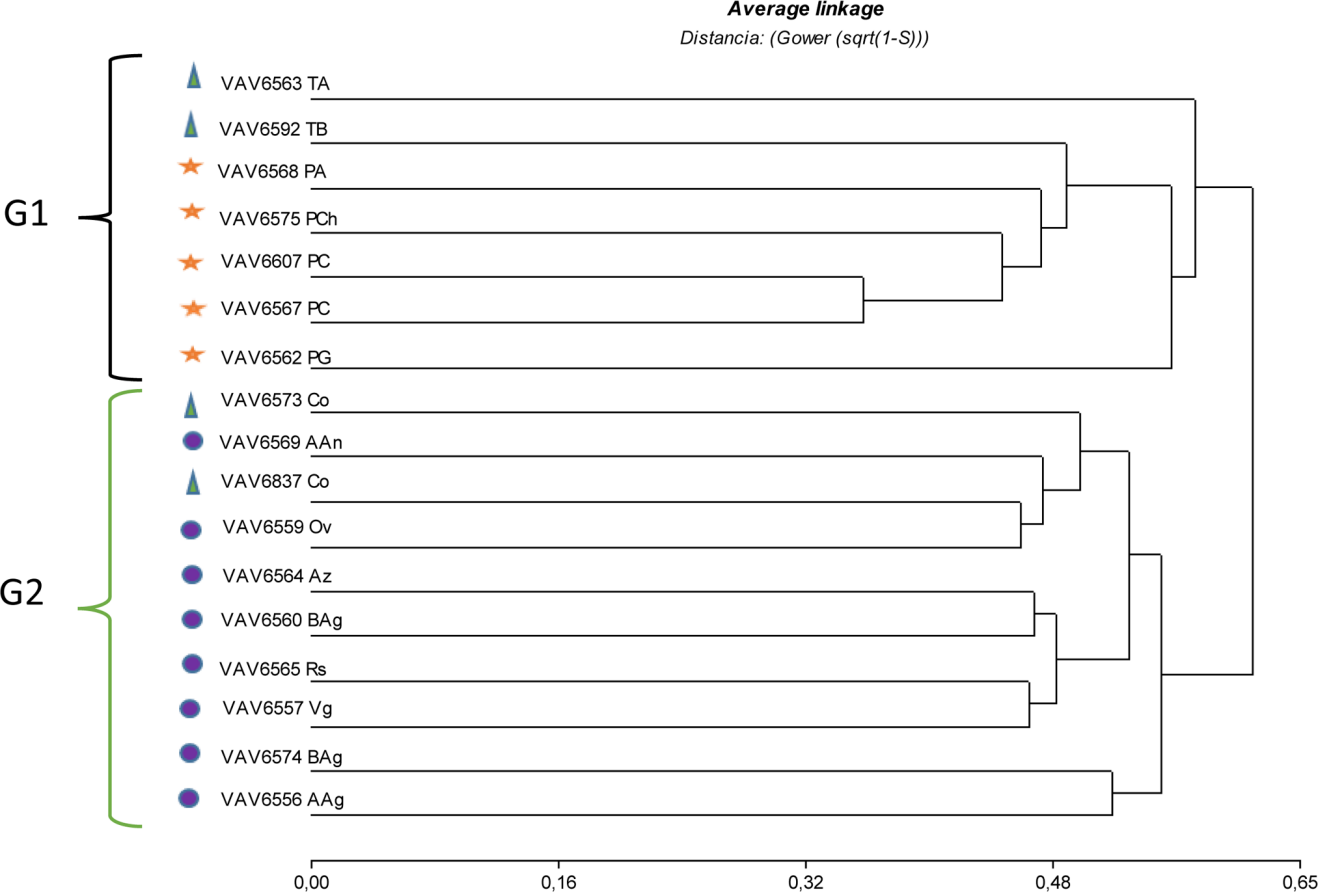
Relationships among Guaranís maize populations (UPGMA). Shapes represented populations with different type of grain. **Ref.** Yellow stars: Popcorn maize populations. Blue circles: Floury populations. Green triangle: Floury grains with corneal periphery (F-P_C_) populations. Yellow stars: Popcorn populations. PCh: Pororó Chico. PG: Pororó Grande. PA: Pipoca Amarillo. PC: Pipoca Colorado. TA: Tupí Amarillo. TB: Tupí Blanco. Az: Azul. Rs: Rosado. Vg: Variegado. AAn: Amarillo. Ancho. AAg: Amarillo Angosto. Co: Colorado. Ov: Overo. Ban: Blanco Ancho. Bag: Blanco Angosto.

The principal components analysis (PCoA) showed that the first 10 axis account for 72% of the total variability, and the first four account for 43% of total variability. Biplots graphs showed that individuals from Floury populations were more closely related within each other than with those of Popcorn populations. The individuals from F showed different distributions than those of Pc populations, showing karyotype differences between individuals belonging to these 2 groups. Individuals from the F-Pc populations showed a wide dispersion (S3 Fig). The UPGMA cluster analysis (cophenetic correlation = 0.746) showed two groups, G1 and G2, at an approximately Gower distance of 0.6. The G1 group included all Pc populations and F-Pc populations of Tupí (VAV6563 and VAV6592). The G2 group included all the F populations and two F-Pc populations of Colorado (VAV6837 and VAV6573) (Fig 4).

## Discussion

The joint analysis of karyotype parameters provides the first thorough cytogenetic characterization of Guaraní Argentinian maize from Northeastern Argentina (NEA). The cytogenetic variability of 20 populations of Guaraní landraces was analyzed. Furthermore, our karyotype results were compared with those previously reported from Northwestern Argentina (NWA) and Central Region of South America maize.

### Karyotype parameters: Intra- and inter-populational variability

In maize Guaraní landraces, high intra- and inter-populational karyotype variability were detected. The inter-populational variation in the number of knobs and percentage of heterochromatin was significant, varying from 8 to 23 knobs and from 5.06% to 20.02%, respectively. Although a significant positive relationship was found between the number of knobs and percentage of heterochromatin, individuals of the same population with the same number of knobs, not always exhibited similar percentages of heterochromatin. This suggests that the percentage of heterochromatin in each individual depends not only in the number but also in the size of knobs, given mainly by the number of copies of the satellite DNA repeats that conform them. Considering all the populations that were analyzed, knobs were observed at 20 different chromosomal positions, with inter-populational differences in their frequencies. Knob positions 6L_2_ and 6L_3_, described in pachytene chromosomes by McClintock *et al*. 1981 [12], were only observed in the VAV6560 population, which was a distinctive karyotype trait this population. It is interesting to note that populations of the same race exhibited similarities in the percentage of heterochromatin as well as in the number and the frequent positions of knobs. In addition, chromosomes with AB10 morphology were detected, with low frequencies (0.1≥ *f* ≤0.40), in only six populations. On this basis it could be concluded that the populations of the Guaraní landraces could be characterized by the set of karyotype parameters presented here, defining for the first time their chromosomal constitution. Therefore, the karyotype parameters could be used as another index of variability and taken into account in maize landraces studies for sure.

The percentage of heterochromatin and the number of knobs did not show a significant correlation with the CV_CL_, A_1_ and M_AC_ indexes. This could be due to the great intra- and inter-populational variability detected in position and size of knobs, and to differences in the content and location of other repetitive sequences scattered throughout the genome. However, the relationship found between the CV_CI_ index and the number of knobs, and the percentage of heterochromatin could be explained because centromeric index and karyotype asymmetry depend on chromosomal heterochromatin distribution. In addition, the amplification of scattered sequences also affects the chromosomal arm size and consequently, the centromeric index [35].

The FISH experiments allowed detecting the sequence composition of each knob. The majority of studied individuals had all their knobs mixed. As the 180-bp hybridization signals were more intense than TR-1 signals, it is possible to infer that the mixed knobs would be composed of greater numbers of 180-bp repeats than TR-1 repeats. It has been proposed that TR-1 arose by duplication and divergence of the 180-bp sequence [15, 16]. Therefore, it is possible that the 180-bp repeat formed knobs before TR-1 appeared. In this scenario, TR-1 might have invaded 180-bp loci, optimizing its chances of being transmitted preferentially through the AB10-mediated meiotic impulse [23, 36]. On the other hand, the sequence composition of each knob detected here did not allow discrimination among Guaraní landraces.

Microsatellite studies and multivariate analysis of morphological, phenological and reproductive traits identified two different genetic groups within the NEA maize, Floury and Popcorn [3, 37]. In the present study, the number of knobs and the percentages of heterochromatin showed that the Popcorn and Tupí populations differ from the Floury populations. In addition, the Floury populations have only six knobs positions exhibiting high knob frequency, whereas F-Pc and Popcorn populations showed a higher number of knob positions with high frequency. The UPGMA and the PcoA analyses supported the differences between the Floury and Popcorn populations at the karyotype level. F-Pc populations exhibited wide dispersion, where the Tupí populations were more closely related to the Popcorn populations. The congruence between the clusters obtained from the analysis of molecular markers, morphological / phenological traits and karyotype parameters confirm that Popcorn and Floury populations from NEA are clearly different genetic groups.

### Relationship with South American Continent landraces

In order to contrast the Horowitz hypothesis of two different centers on diversification of Argentinian landraces, NEA and NWA [2], our results were compared with previous cytogenetic studies from NWA landraces [5, 6, 13, 18]. Important karyotype differences were observed between maize from both regions, the maximum number of knobs in NEA (from 8 to 23 knobs) was higher than that reported for NWA landraces (from 5 to 19 knobs). There were also differences at the most frequent knob chromosome positions, as NEA had a higher number of knobs than those reported in NWA landraces, where only 6-Sat and 9S were founded as the most frequent positions [6]. In our study we detected, in low frequency, chromosomes with AB10 morphology in six NEA landraces cultivated from 98 to 600 m.a.s.l., but these chromosomes were not described in NWA that grown up to 3900 m.a.s.l [5, 6]. These results are in agreement with the reported negative correlation between AB10 presence and altitude of cultivation associated to the selection for faster growth and against larger genome size at higher altitudes [21, 36]. Moreover, Bs has not been detected in the Guaraní populations, but in NWA landraces numerical polymorphism (0–8) and frequencies up to 100% have been reported [5, 6, 13]. It could be concluded that these results allow discrimination between the NEA and NWA maize landraces, supporting the Horowitz hypothesis.

McClintock *et al*. 1981 [12], based on the knobs karyotype constitution from American races, proposed that maize from Northern South America extended to Eastern South America, while the races found in the Central Andean highlands extended along Western South America. In addition, these authors observed an almost total absence of B chromosomes for the eastern region of South America, and high frequency of Bs in the western region of South America. This hypothesis has been later supported by microsatellite analysis [4, 37–39]. Therefore, the karyotype differences between the NWA and the NEA landraces reported here support the hypothesis that the Southern South American maize would have been introduced from two routes, a highland route along the Andes and a lowland route along the Northeastern coast.

The relationship between NEA landraces and those of the lowlands in the American Continent was determined by analysis of molecular markers, revealing the existence of three different genetic groups: Tropical lowland maize, Floury maize, and Popcorn maize from NEA [4]. Also, it was proposed that Guaraní maize would be related to those of the Central Region described by McClintock *et al*. 1981 [12]. The results obtained here were compared with the maize from the Central Region. In this region 12 positions of knobs (1S, 2L, 3L, 4L, 5L, 6L_2_, 6L_3_, 7L, 8L_1_, 8L_2_, 9S and 9L) were reported, while in the Guaraní landraces the majority of those positions plus1L, 2S, 3S, 4S, 5S, 6-Sat, 7S, 8S and 10L were detected. This greater number of knob positions that we found in Guaraní maize could be due to the higher resolution cytogenetic techniques (DAPI banding and FISH) applied in the present work. In NEA and Central Region the B chromosomes were absent and AB10 chromosomes were reported with low frequency. These cytogenetic similarities allow us to infer a correspondence between maize from both regions. This fact, together with the significant differences founded between the NEA and NWA landraces support the hypothesis that maize was introduced in two ways in South America.

In summary, the joint analysis of karyotype parameters allowed us the first thorough karyotypic characterization of Guaraní maize from NEA and to identify two different karyotype groups, the Flourys and Popcorns. Furthermore, this work highlights the remarkable karyotype differentiation with NWA maize. The cytogenetic characterization of landraces from NEA contributes to the knowledge of the genetic variability of the Argentinian native maize and postulates this germoplasm as genetic resource to improve important agronomic traits.

## Supporting information

S1 Fig *Consensus* idiograms and knob position histograms of Floury Guaraní maize populations of NEA, where the position, average size and frequency of each knob are indicate. (**A)** VAV6564, Azul. (**B)** VAV6569, Amarillo Ancho. (**C)** VAV6556, Amarillo Angosto. (**D)** VAV6560, Blanco Ancho. (**E)** VAV6574, Blanco Angosto. (**F)** VAV6559, Overo. (**G)** VAV6565, Rosado. (**H)** VAV6557, Variegado. **Ref.** The average size of the knobs is represented by the size of the bands on the idiograms (S_K_, M_K_ and L_K_). The black blocks/ bars indicate the most frequent positions (*f* ≥0.6). The white blocks / bars show positions of less frequency (*f* <0.6). The size of each knob was estimated in relation to the chromosome length: small knobs (S_K_) ≤ 10%, medium knobs (M_K_) between 10% and 20%, and large knobs (L_K_) ≥ 20% of chromosome length. Cr: chromosomal pair. L: long arm. S: short arm. Sat: satellite region.
(TIF)

S2 Fig *Consensus* idiograms and knob position histograms of Popcorn and F-Pc Guaraní maize populations of NEA, where the position, average size and frequency of each knob are indicated. (**A)** VAV6568, Pipoca Amarillo. (**B)** VAV6567, Pipoca Colorado. (**C)** VAV6607, Pipoca Colorado. (**D)** VAV6575, Pororó Chico. (**E)** VAV6562, Pororó Grande. (**F)** VAV6573, Colorado. (**G)** VAV6837, Colorado. (**H)** VAV6563, Tupí Amarillo. (**I)** VAV6592, Tupí Blanco. **Ref.** The average size of the knobs is represented by the size of the bands on the idiograms (S_K_, M_K_ and L_K_). The black blocks/ bars indicate the most frequent positions (*f* ≥0.6). The white blocks / bars show positions of less frequency (*f* <0.6). The size of each knob was estimated in relation to the chromosome length: small knobs (S_K_) ≤ 10%, medium knobs (M_K_) between 10% and 20%, and large knobs (L_K_) ≥ 20% of chromosome length. Cr: chromosomal pair. L: long arm. S: short arm. Sat: satellite region.
(TIF)

S3 Fig Distribution of the individuals from Guaranís maize populations, on the first four axis of the principal coordinates analysis—PCoA. (A). Biplot axis 2 vs. axis 1, total variability 27.6.0%. (B). Biplot axis 3 vs. axis 1, total variability 27.5%. (C). Biplot axis 4 vs. axis 1, total variability 26.9%. Colors representing individuals belong to maize populations with different types of grains. The percentages in the axis labels represent the percentages of variation explained by the principal coordinates. Ref. Blue circles: individuals of Popcorn (Pc) maize populations. Yellow circles: individuals of Floury (F) maize populations. Green circles: individuals of Floury grains with corneal periphery (F-Pc) maize populations.
(TIF)

S1 Table Mean size of knobs and average centromeric index (CI) values of each chromosomal pair for the studied Guaraní populations. **Ref.** S_K_: small knobs (≤ 10% of the chromosome length). M_K_: medium knobs (between 10% and 20% of the chromosome length). L_K_: large knobs (20% > of the chromosome length). SD: standard deviation. Cr: chromosomal pair. L: long arm. S: short arm. Sat: satellite region.
(DOCX)

S2 Table Analysis of relationships among the karyotype parameters (Spearman coefficient). **Ref.** CV_CI_: Coefficient of variation of centromeric indexes; CV_CL_: Coefficient of variation of chromosome length; A1: intracromosomal asymmetry index; M_CA_; Mean chromosomal asymmetry index; TCL: Total chromosome length. Boldness shows significant correlations between parameters (p <0.05).
(DOCX)
