# Supplementary figures and images for: Exploring karyotype diversity of Argentinian Guaraní maize landraces: Relationship among South American maize
Source: PLoS One. 2018 Jun 7;13(6):e0198398. doi: 10.1371/journal.pone.0198398 (PMC5991688; doi:10.1371/journal.pone.0198398)

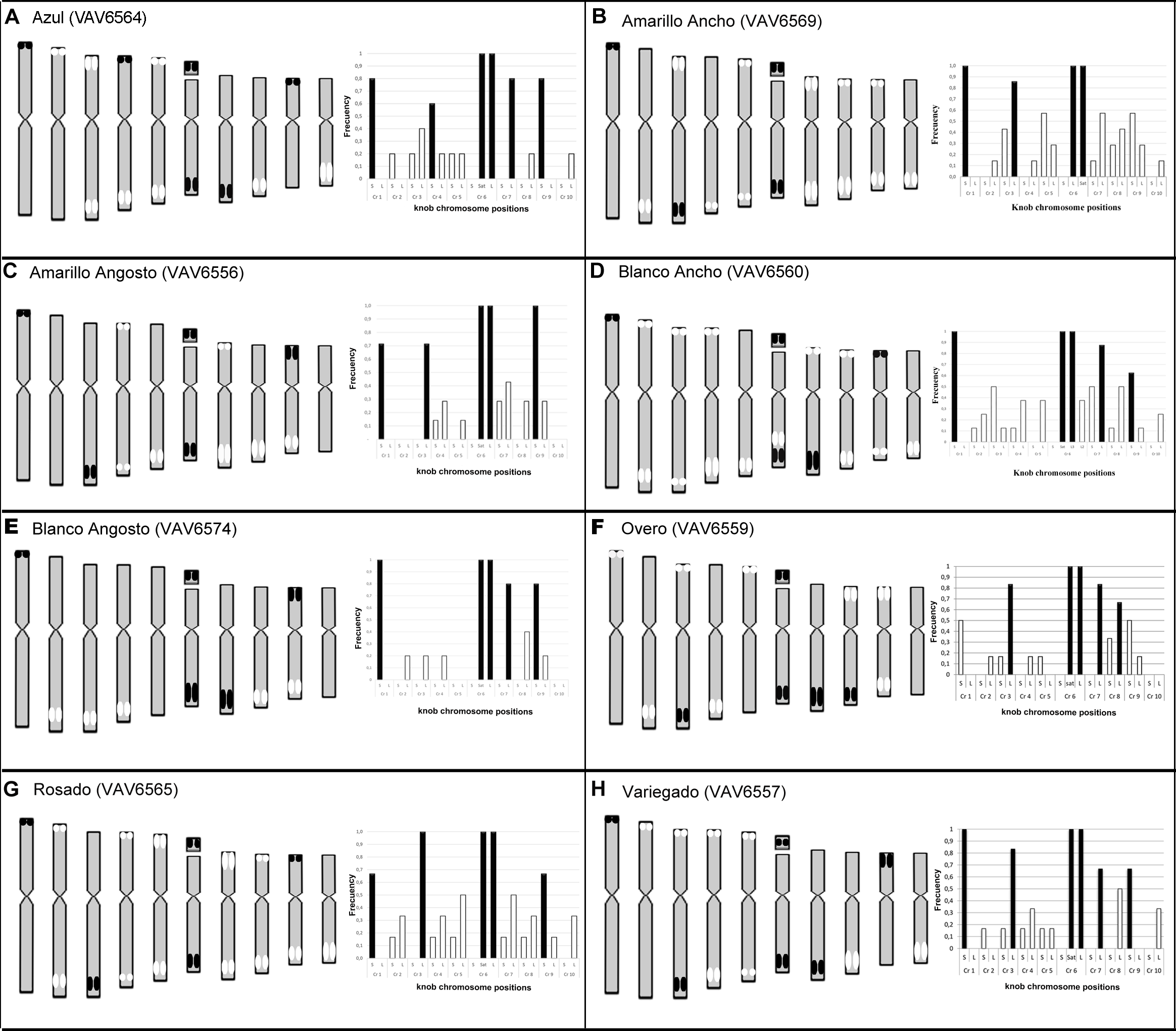

Supplement: S1 Fig — (A) VAV6564, Azul. (B) VAV6569, Amarillo Ancho. (C) VAV6556, Amarillo Angosto. (D) VAV6560, Blanco Ancho. (E) VAV6574, Blanco Angosto. (F) VAV6559, Overo. (G) VAV6565, Rosado. (H) VAV6557, Variegado. Ref. The average size of the knobs is represented by the size of the bands on the idiograms (SK, MK and LK). The black blocks/ bars indicate the most frequent positions (f ≥0.6). The white blocks / bars show positions of less frequency (f <0.6). The size of each knob was estimated in relation to the chromosome length: small knobs (SK) ≤ 10%, medium knobs (MK) between 10% and 20%, and large knobs (LK) ≥ 20% of chromosome length. Cr: chromosomal pair. L: long arm. S: short arm. Sat: satellite region. (TIF) [file pone.0198398.s001.tif]

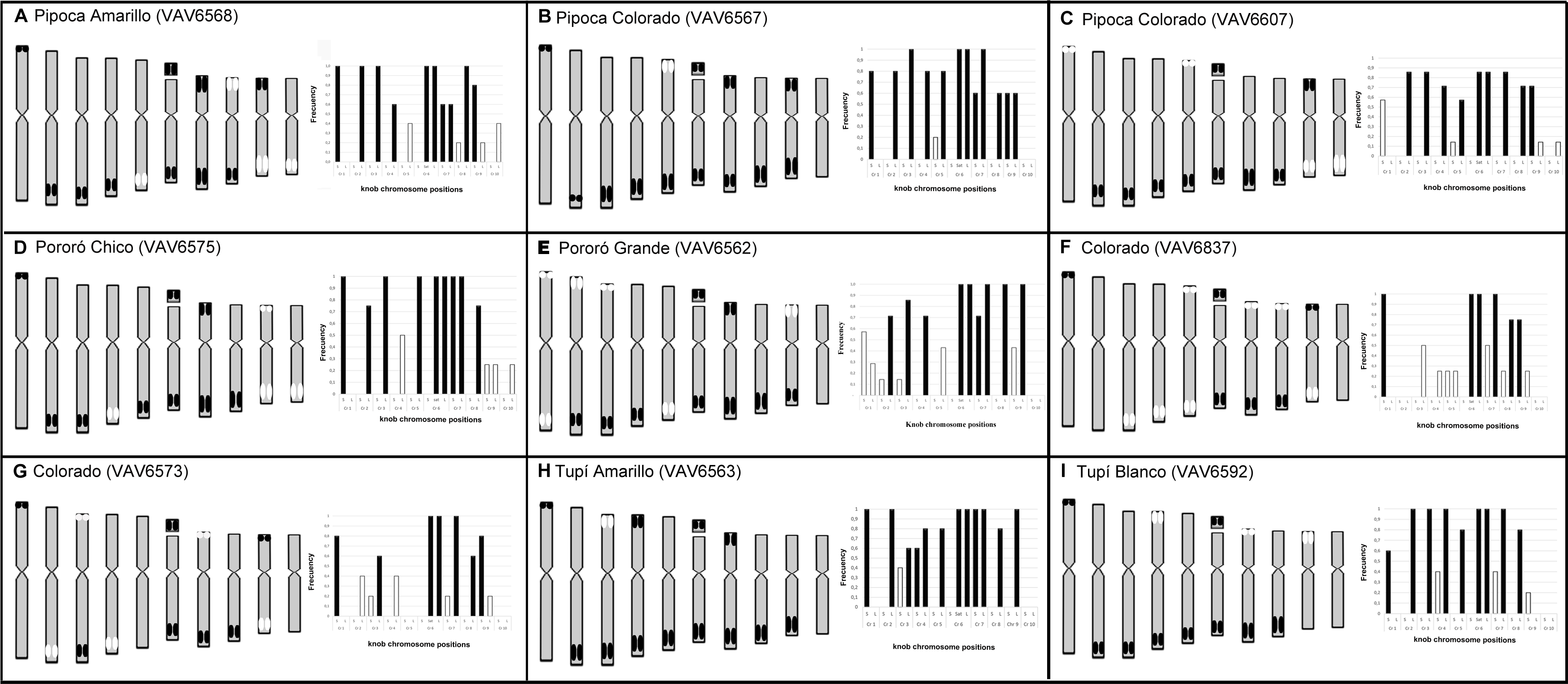

Supplement: S2 Fig — (A) VAV6568, Pipoca Amarillo. (B) VAV6567, Pipoca Colorado. (C) VAV6607, Pipoca Colorado. (D) VAV6575, Pororó Chico. (E) VAV6562, Pororó Grande. (F) VAV6573, Colorado. (G) VAV6837, Colorado. (H) VAV6563, Tupí Amarillo. (I) VAV6592, Tupí Blanco. Ref. The average size of the knobs is represented by the size of the bands on the idiograms (SK, MK and LK). The black blocks/ bars indicate the most frequent positions (f ≥0.6). The white blocks / bars show positions of less frequency (f <0.6). The size of each knob was estimated in relation to the chromosome length: small knobs (SK) ≤ 10%, medium knobs (MK) between 10% and 20%, and large knobs (LK) ≥ 20% of chromosome length. Cr: chromosomal pair. L: long arm. S: short arm. Sat: satellite region. (TIF) [file pone.0198398.s002.tif]

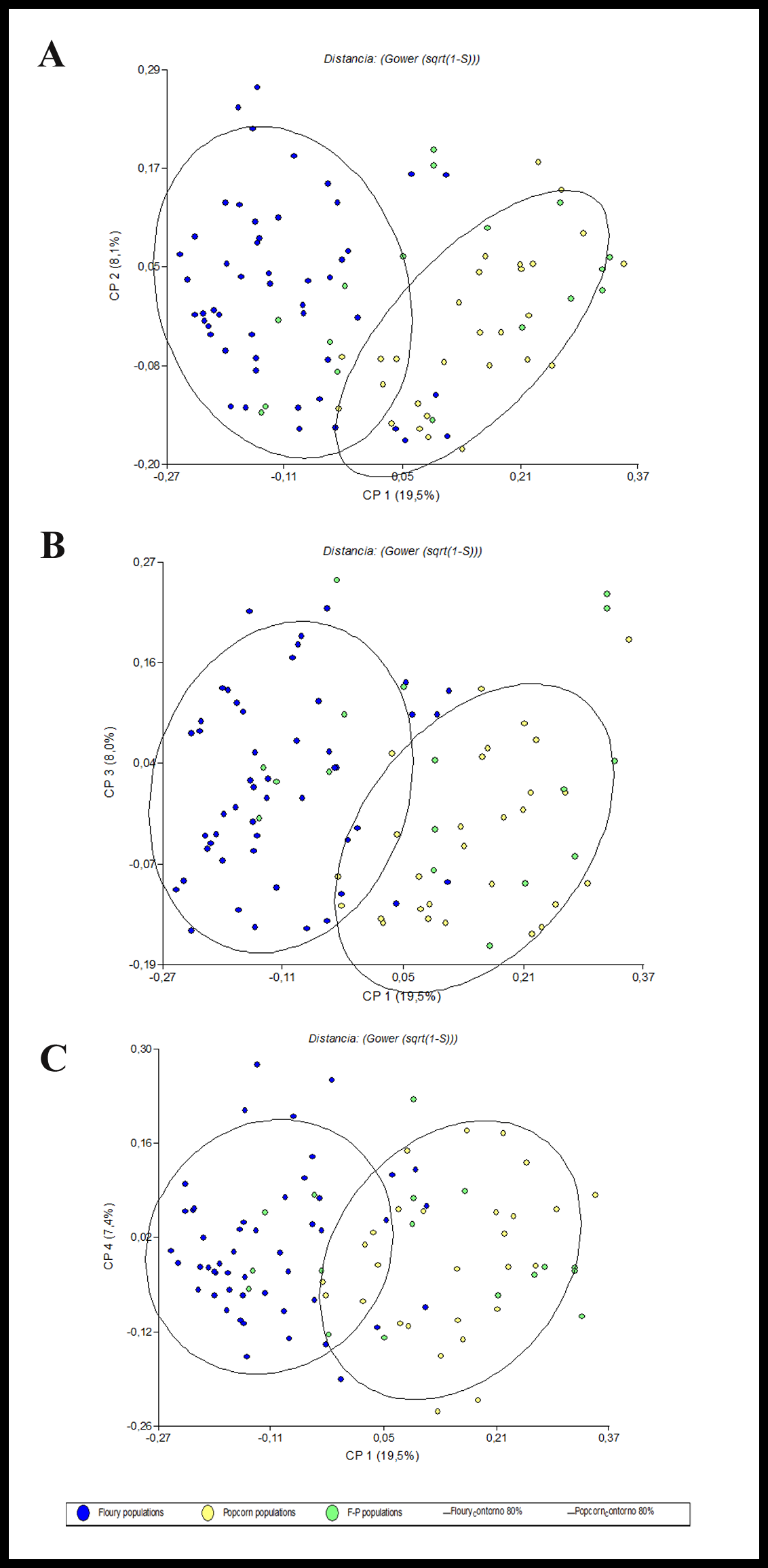

Supplement: S3 Fig — (A). Biplot axis 2 vs. axis 1, total variability 27.6.0%. (B). Biplot axis 3 vs. axis 1, total variability 27.5%. (C). Biplot axis 4 vs. axis 1, total variability 26.9%. Colors representing individuals belong to maize populations with different types of grains. The percentages in the axis labels represent the percentages of variation explained by the principal coordinates. Ref. Blue circles: individuals of Popcorn (Pc) maize populations. Yellow circles: individuals of Floury (F) maize populations. Green circles: individuals of Floury grains with corneal periphery (F-Pc) maize populations. (TIF) [file pone.0198398.s003.tif]
