# Supplementary material for: Exploring karyotype diversity of Argentinian Guaraní maize landraces: Relationship among South American maize
Source: PLoS One. 2018 Jun 7;13(6):e0198398. doi: 10.1371/journal.pone.0198398 (PMC5991688; doi:10.1371/journal.pone.0198398)
Supplement: S1 Table — Ref. SK: small knobs (≤ 10% of the chromosome length). MK: medium knobs (between 10% and 20% of the chromosome length). LK: large knobs (20% > of the chromosome length). SD: standard deviation. Cr: chromosomal pair. L: long arm. S: short arm. Sat: satellite region. (DOCX) [file pone.0198398.s004.docx]

| **Chromosome**  **pair** | | **1 Cr** | | **2 Cr** | | | **3 Cr** | | **4 Cr** | | **5 Cr** | | **6 Cr** | | | **7 Cr** | | **8 Cr** | | **9 Cr** | | **10 Cr** | | |
| --- | --- | --- | --- | --- | --- | --- | --- | --- | --- | --- | --- | --- | --- | --- | --- | --- | --- | --- | --- | --- | --- | --- | --- | --- |
| **Populations/**  **VAV** | **Positions** | **S** | **L** | **S** | **L** | | **S** | **L** | **S** | **L** | **S** | **L** | **S** | **Sat** | **L** | **S** | **L** | **S** | **L** | **S** | **L** | **S** | **L** | |
| **VAV 6557** | **Size of knob** | **S_K_** | **_--_** | **S_K_** | **_--_** | | **S_K_** | **M_K_** | **S_K_** | **M_K_** | **S_K_** | **S_K-_M_K_** | **_--_** | **S_K-_M_K_** | **M_K-_L_K_** | **_--_** | **M_K-_L_K_** | **_--_** | **M_K-_L_K_** | **M_K_** | **_--_** | **_--_** | **M_K-_L_K_** | |
|  | **CI ± SD** | 0,45 ± 0,03 | | 0,42 ± 0,04 | | | 0,39 ± 0,02 | | 0,40 ± 0,05 | | 0,43 ± 0,04 | | 0,35 ± 0,04 | | | 0,35 ± 0,08 | | 0,35 ± 0,05 | | 0,37 ± 0,05 | | 0,37 ± 0,05 | | |
| **VAV6564** | **Size of knob** | **S_K_** | **_--_** | **S_K_** | **_--_** | | **M_K_** | **S_K-_M_K_** | **S_K_** | **M_K_** | **S_K_** | **M_K_** | **_--_** | **S_K-_M_K_** | **M_K_** |  | **S_K-_M_K_** | **_--_** | **M_K_** | **S_K_** | **_--_** | **_--_** | **L_K_** | |
|  | **CI ± SD** | 0,44 ± 0,03 | | 0,43 ±0,04 | | | 0,39 ±0,07 | | 0,43 ± 0,01 | | 0,43 ±0,03 | | 0,32 ±0,02 | | | 0,36 ±0,06 | | 0,39 ±0,07 | | 0,42 ±0,07 | | 0,41 ±0,06 | | |
| **VAV6556** | **Size of knob** | **S_K_** | **_--_** | **_--_** | **_--_** | | **_--_** | **M_K_** | **S_K_** | **S_K_** | **_--_** | **M_K_** | **_--_** | **M_K_** | **M_K-_L_K_** | **S_K_** | **M_K-_L_K_** | **_--_** | **M_K-_L_K_** | **S_K-_M_K_** | **M_K_** | **_--_** | **_--_** | |
|  | **CI ± SD** | 0,42 ± 0,05 | | 0,42 ± 0,05 | | | 0,38 ± 0,07 | | 0,44 ± 0,04 | | 0,44 ± 0,03 | | 0,36 ± 0,05 | | | 0,36 ± 0,04 | | 0,41 ± 0,06 | | 0,45 ± 0,04 | | 0,44 ± 0,04 | | |
| **VAV6569** | **Size of knob** | **S_K_** |  | **_--_** | **M_K_** | | **M_K-_L_K_** | **M_K_** | **_--_** | **S_K_** | **S_K_** | **S_K_** | **_--_** | **M_K_** | **M_K_** | **M_K_** | **M_K-_L_K_** | **S_K_** | **M_K_** | **S_K_** | **M_K_** | **_--_** | **M_K_** | |
|  | **CI ± SD** | 0,45 ± 0,02 | | 0,41 ± 0,02 | | | 0,37 ± 0,04 | | 0,46 ± 0,03 | | 0,43 ± 0,03 | | 0,33 ± 0,02 | | | 0,37 ± 0,08 | | 0,35 ± 0,05 | | 0,39 ± 0,04 | | 0,38 ± 0,04 | | |
| **VAV6574** | **Size of knob** | **S_K_** | **_--_** | **_--_** | **M_K_** | | **_--_** | **M_K_** | **_--_** | **M_K_** | **_--_** | **_--_** | **_--_** | **M_K-_L_K_** | **M_K-_L_K_** | **_--_** | **M_K-_L_K_** | **_--_** | **M_K_** | **S_K-_M_K_** | **S_K-_M_K_** | **_--_** | **_--_** | |
|  | **CI ± SD** | 0,45 ± 0,06 | | 0,40 ± 0,04 | | | 0,40 ± 0,06 | | 0,41 ± 0,03 | | 0,41 ± 0,06 | | 0,34 ± 0,04 | | | 0,34 ± 0,06 | | 0,35 ± 0,05 | | 0,41 ± 0,05 | | 0,38 ± 0,04 | | |
| **VAV6560** | **Size of knob** | **S_K_** | **_--_** | **S_K_** | **S_K-_M_K_** | | **S_K_** | **S_K_** | **S_K_** | **M_K-_L_K_** | **_--_** | **M_K_** | **_--_** | **M_K_** | **M_K_** | **S_K-_M_K_** | **M_K-_L_K_** | **S_K_** | **M_K_** | **S_K-_M_K_** | **S_K_** | **_--_** | **M_K_** | |
|  | **CI ± SD** | 0,46 ± 0,02 | | 0,41 ± 0,03 | | | 0,40 ± 0,05 | | 0,42 ± 0,04 | | 0,43 ± 0,04 | | 0,34 ± 0,05 | | | 0,37 ± 0,06 | | 0,39 ± 0,06 | | 0,37 ± 0,03 | | 0,37 ± 0,07 | | |
| **VAV6565** | **Size of knob** | **S_K_** | **_--_** | **S_K_** | **M_K_** | | **_--_** | **M_K_** | **S_K_** | **S_K-_M_K_** | **M_K_** | **M_K_** | **_--_** | **M_K_** | **M_K-_L_K_** | **L_K_** | **M_K-_L_K_** | **S_K-_M_K_** | **M_K_** | **S_K-_M_K_** | **M_K_** | **_--_** | **L_K_** | |
|  | **CI ± SD** | 0,46 ± 0,02 | | 0,41 ± 0,03 | | | 0,41 ± 0,03 | | 0,45 ± 0,03 | | 0,38 ± 0,07 | | 0,34 ± 0,03 | | | 0,39 ± 0,05 | | 0,36 ± 0,06 | | 0,39 ± 0,05 | | 0,40 ± 0,05 | | |
| **VAV6559** | **Size of knob** | **S_K_** | **_--_** | **_--_** | **M_K_** | | **S_K_** | **M_K_** | **_--_** | **M_K_** | **S_K_** | **_--_** | **_--_** | **S_K-_M_K_** | **S_K-_M_K_** | **_--_** | **M_K-_L_K_** | **M_K_** | **M_K-_L_K_** | **S_K-_M_K_** | **M_K-_L_K_** | **_--_** | **_--_** | |
|  | **CI ± SD** | 0,45 ± 0,04 | | 0,42 ± 0,02 | | | 0,45 ± 0,02 | | 0,41 ± 0,03 | | 0,42 ± 0,07 | | 0,34 ± 0,04 | | | 0,35 ± 0,04 | | 0,36 ± 0,07 | | 0,38 ± 0,04 | | 0,37 ± 0,03 | | |
| **VAV6562** | **Size of knob** | **S_K_** | **M_K_** | **M_K-_L_K_** | | **S_K-_M_K_** | **S_K_** | **M_K_** |  | **M_K_** |  | **M_K_** | **_--_** | **M_K_** | **M_K-_L_K_** | **M_K-_L_K_** | **M_K-_L_K_** |  | **M_K-_L_K_** | **M_K-_L_K_** | **M_K_** | **_--_** | **_--_** | |
|  | **CI ± SD** | 0,43 ± 0,04 | | 0,40 ± 0,03 | | | 0,40 ± 0,06 | | 0,39 ± 0,07 | | 0,44 ± 0,04 | | 0,31 ± 0,06 | | | 0,38 ± 0,07 | | 0,34 ± 0,07 | | 0,39 ± 0,07 | | 0,40 ± 0,05 | | |
| **VAV6575** | **Size of knob** | **S_K_** | **_--_** |  | **M_K_** | |  | **M_K_** |  | **M_K_** |  | **M_K_** | **_--_** | **S_K-_M_K_** | **S_K-_M_K_** | **M_K_** | **M_K-_L_K_** | **_--_** | **M_K_** | **S_K_** | **M_K-_L_K_** | **_--_** | **L_K_** | |
|  | **CI ± SD** | 0,45 ± 0,02 | | 0,41 ± 0,02 | | | 0,37 ± 0,04 | | 0,46 ± 0,03 | | 0,43 ± 0,03 | | 0,33 ± 0,02 | | | 0,37 ± 0,08 | | 0,35 ± 0,05 | | 0,39 ± 0,04 | | 0,38 ± 0,04 | | |
| **VAV6567** | **Size of knob** | **S_K_** | **_--_** | **_--_** | **S_K-_M_K_** | | **_--_** | **M_K_** | **_--_** | **M_K-_L_K_** | **L_K_** | **M_K-_L_K_** | **_--_** | **M_K_** | **M_K_** | **S_K-_M_K_** | **M_K-_L_K_** | **_--_** | **M_K-_L_K_** | **S_K-_M_K_** | **M_K-_L_K_** | **_--_** | **_--_** | |
|  | **CI ± SD** | 0,45 ± 0,03 | | 0,42 ± 0,05 | | | 0,37 ± 0,04 | | 0,43 ± 0,04 | | 0,38 ± 0,08 | | 0,29 ± 0,05 | | | 0,36 ± 0,09 | | 0,38 ± 0,04 | | 0,41 ± 0,02 | | 0,39 ± 0,03 | | |
| **VAV6607** | **Size of knob** | **S_K-_M_K_** | **_--_** | **_--_** | **M_K-_L_K_** | | **_--_** | **M_K-_L_K_** | **_--_** | **M_K-_L_K_** | **S_K_** | **M_K_** | **_-_** | **M_K_** | **M_K_-L_K_** | **_--_** | **M_K-_L_K_** | **_--_** | **M_K-_L_K_** | **M_K-_L_K_** | **M_K_** | **_--_** | **L_K_** | |
|  | **CI ± SD** | 0,45 ± 0,01 | | 0,39 ± 0,05 | | | 0,37 ± 0,03 | | 0,43 ± 0,04 | | 0,46 ± 0,02 | | 0,34 ± 0,03 | | | 0,35 ± 0,05 | | 0,32 ± 0,04 | | 0,41 ± 0,06 | | 0,37 ± 0,06 | | |
| **VAV6568** | **Size of knob** | **S_K_** | **_--_** | **_--_** | **M_K-_L_K_** | | **_--_** | **M_K-_L_K_** | **_--_** | **M_K-_L_K_** | **_--_** | **M_K_** | **_-_** | **M_K-_L_K_** | **M_K-_L_K_** | **M_K-_L_K_** | **M_K-_L_K_** | **M_K_** | **M_K-_L_K_** | **M_K-_L_K_** | **L_K_** | **_--_** | **M_K-_L_K_** | |
|  | **CI ± SD** | 0,45 ± 0,02 | | 0,42 ± 0,02 | | | 0,38 ± 0,05 | | 0,43 ± 0,03 | | 0,44 ± 0,03 | | 0,30 ± 0,04 | | | 0,34 ± 0,08 | | 0,38 ± 0,07 | | 0,41 ± 0,06 | | 0,40 ± 0,04 | | |
| **VAV6563** | **Size of knob** | **S_K-_M_K_** | _--_ | **_--_** | **M_K-_L_K_** | | **M_K_** | **M_K-_L_K_** | **M_K-_L_K_** | **M_K-_L_K_** | **_--_** | **M_K-_L_K_** | **_--_** | **M_K_** | **M_K-_L_K_** | **M_K-_L_K_** | **M_K-_L_K_** | **_--_** | **M_K-_L_K_** | **_--_** | **M_K-_L_K_** | **_--_** | **_--_** | |
|  | **CI ± SD** | 0,46 ± 0,02 | | 0,39 ±0,04 | | | 0,37 ±0,05 | | 0,42 ± 0,04 | | 0,39 ±0,06 | | 0,33 ±0,05 | | | 0,40 ±0,06 | | 0,38 ±0,07 | | 0,39 ±0,08 | | 0,40 ±0,05 | | |
| **VAV6592** | **Size of knob** | **S_K_** | **_--_** | **_--_** | **M_K-_L_K_** | |  | **M_K-_L_K_** | **M_K_** | **M_K-_L_K_** | **_--_** | **M_K-_L_K_** | **_--_** | **S_K-_M_K_** | **M_K_** | **S_K_** | **M_K-_L_K_** | **_--_** | **L_K_** | **_--_** | **L_K_** | **_--_** | | **_--_** |
|  | **CI ± SD** | 0,46 ± 0,03 | | 0,45 ±0,03 | | | 0,36 ±0,02 | | 0,43 ± 0,03 | | 0,44 ±0,03 | | 0,33 ±0,03 | | | 0,35 ±0,04 | | 0,38 ±0,05 | | 0,41 ±0,06 | | 0,43 ±0,04 | | |
| **VAV6573** | **Size of knob** | **S_K-_M_K_** | **_--_** | **_--_** | **M_K_** | | **S_K_** | **M_K_** | **_--_** | **S_K-_M_K_** | **_--_** | **_--_** | **_--_** | **M_K_** | **M_K_** | **S_K_** | **M_K-_L_K_** | **_--_** | **M_K_** | **S_K-_M_K_** | **M_K_** | **_--_** | | **_--_** |
|  | **CI ± SD** | 0,46 **±** 0,03 | | 0,43 **±** 0,04 | | | 0,43 **±** 0,04 | | 0,44 **±** 0,02 | | 0,44 **±** 0,02 | | 0,31 **±** 0,04 | | | 0,37 **±** 0,07 | | 0,39 **±** 0,06 | | 0,40 **±** 0,03 | | 0,39 **±** 0,05 | | |
| **VAV6837** | **Size of knob** | **S_K-_M_K_** | **_--_** | **_--_** | **_--_** | | **_--_** | **M_K_** |  | **M_K_** | **S_K_** | **M_K_** | **_--_** | **S_K-_M_K_** | **M_K-_L_K_** | **S_K_** | **M_K-_L_K_** | **S_K_** | **M_K_** | **S_K-_M_K_** | **M_K_** | **_--_** | | **_--_** |
|  | **CI ± SD** | 0,47 **±** 0,03 | | 0,46 **±** 0,05 | | | 0,38 **±** 0,06 | | 0,42 **±** 0,03 | | 0,42 **±** 0,03 | | 0,36 **±** 0,07 | | | 0,32 **±** 0,03 | | 0,41 **±** 0,05 | | 0,46 **±** 0,02 | | 0,39 **±** 0,08 | | |

**Supplementary Table 1.** Mean size of the knobs of each Chromosomal position and mean Centromeric index (CI) of each Chromosomal pair for the studied Guaraní populations.
