# Supplementary material for: Exploring karyotype diversity of Argentinian Guaraní maize landraces: Relationship among South American maize
Source: PLoS One. 2018 Jun 7;13(6):e0198398. doi: 10.1371/journal.pone.0198398 (PMC5991688; doi:10.1371/journal.pone.0198398)
Supplement: S2 Table — Ref. CVCI: Coefficient of variation of centromeric indexes; CVCL: Coefficient of variation of chromosome length; A1: intracromosomal asymmetry index; MCA; Mean chromosomal asymmetry index; TCL: Total chromosome length. Boldness shows significant correlations between parameters (p <0.05). (DOCX) [file pone.0198398.s005.docx]

**S2 Table**. Analysis of relationships among the karyotype parameters (Spearman coefficient).

| **Variable (1)** | **Variable (2)** | **n** | **Spearman coefficients** | **P-values** |
| --- | --- | --- | --- | --- |
| **CV_CI_** | Percentages of knob  heterochromatin | 97 | **0.3** | **≤0.0001** |
|  | Numbers of knobs | 97 | **0.26** | **0.01** |
| **CV_CL_** | Percentages of knob  heterochromatin | 97 | 0.00 | 0.97 |
|  | Number of knobs | 97 | 0.1 | 0.33 |
| **M_AC_** | Percentages of knob  heterochromatin | 95 | 0.16 | 0.12 |
|  | Numbers of knobs | 95 | 0.08 | 0.45 |
| **TCL** | Percentages of knob  heterochromatin | 97 | -0.19 | 0.07 |
|  | Numbers of knobs | 97 | -0.06 | 0.58 |
| **Number of knobs** | Percentages of knob  heterochromatin | 97 | **0.88** | **≤0.0001** |
| **A1** | Percentages of knob  heterochromatin | 95 | 0.11 | 0.27 |
|  | Numbers of knobs | 95 | 0.03 | 0.75 |
